# SWATH-MS analysis of cerebrospinal fluid to generate a robust battery of biomarkers for Alzheimer’s disease

**DOI:** 10.1038/s41598-020-64461-y

**Published:** 2020-05-04

**Authors:** Sun Ah Park, Jin Myung Jung, Jun Sung Park, Jeong Ho Lee, Bumhee Park, Hyung Jun Kim, Jeong-Ho Park, Won Seok Chae, Jee Hyang Jeong, Seong Hye Choi, Je-Hyun Baek

**Affiliations:** 1 Lab for Neurodegenerative Dementia, Department of Anatomy and Department of Neurology, Ajou University School of Medicine, Suwon, 16499 Republic of Korea; 2 Neuroscience Graduate Program, Department of Biomedical Sciences, Ajou University Graduate School of Medicine, Suwon, 16499 Republic of Korea; 3 Department of Data Science, The University of Suwon, Hwaseong, 18323 Republic of Korea; 4 Biomedical Science and Engineering Interdisciplinary Program, Korea Advanced Institute of Science and Technology (KAIST), Daejeon, 34141 Republic of Korea; 5 Graduate School of Medical Science and Engineering, Korea Advanced Institute of Science and Technology (KAIST), Daejeon, 34141 Republic of Korea; 6 Department of Biomedical Informatics, Ajou University School of Medicine, Suwon, 16499 Republic of Korea; 7 Office of Biostatistics, Ajou Research Institute for Innovative Medicine, Ajou University Medical Center, Suwon, 16499 Republic of Korea; 8 Department of Neurology Soonchunhyang University Bucheon Hospital, Bucheon, 14584 Republic of Korea; 9 Department of Anesthesiology, Soonchunhyang University Bucheon Hospital, Bucheon, 14584 Republic of Korea; 10 Department of Neurology, Ewha Womans University Mokdong Hospital, Seoul, 07985 Republic of Korea; 11 Department of Neurology, Inha University School of Medicine, Incheon, 22332 Republic of Korea; 12 R&D Center for Clinical Mass Spectrometry, Seegene Medical Foundation, Seoul, 04805 Republic of Korea

**Keywords:** Alzheimer's disease, Dementia

## Abstract

Cerebrospinal fluid (CSF) Aβ42 and tau protein levels are established diagnostic biomarkers of Alzheimer’s disease (AD). However, their inadequacy to represent clinical efficacy in drug trials indicates the need for new biomarkers. Sequential window acquisition of all theoretical fragment ion spectra (SWATH)-based mass spectrometry (MS) is an advanced proteomic tool for large-scale, high-quality quantification. In this study, SWATH-MS showed that VGF, chromogranin-A, secretogranin-1, and opioid-binding protein/cell adhesion molecule were significantly decreased in 42 AD patients compared to 39 controls, whereas 14-3-3ζ was increased (FDR < 0.05). In addition, 16 other proteins showed substantial changes (FDR < 0.2). The expressions of the top 21 analytes were closely interconnected, but were poorly correlated with CSF Aβ42, tTau, and pTau181 levels. Logistic regression analysis and data mining were used to establish the best algorithm for AD, which created novel biomarker panels with high diagnostic value (AUC = 0.889 and 0.924) and a strong correlation with clinical severity (all p < 0.001). Targeted proteomics was used to validate their usefulness in a different cohort (n = 36) that included patients with other brain disorders (all p < 0.05). This study provides a list of proteins (and combinations thereof) that could serve as new AD biomarkers.

## Introduction

Cerebrospinal fluid (CSF) levels of Aβ1–42 (Aβ42), total Tau (tTau), and phosphorylated Tau181 (pTau181) are diagnostic markers of Alzheimer’s disease (AD)^1,2^. These biomarkers are currently used to confirm the core pathology of AD, i.e., Aβ and tau pathology, in deceased patients while they are alive^3^, and are also monitored in AD therapeutic trials^4^. However, accumulating evidence has demonstrated a lack of utility of these markers after clinical onset of AD due to early concentration plateaus^5^ and poor reflection of the clinical benefits of therapeutics that target them^6^. CSF proteins related to synaptic function, neurodegeneration, inflammation, and Aβ metabolism have recently been suggested as supplementary biomarkers^7^; however, they have not yet been clinically adopted.

Advances in proteomic techniques have allowed the characterisation and quantification of several CSF proteins related to AD^8^. Targeted mass spectrometric methods, such as multiple reaction monitoring (MRM) and parallel reaction monitoring (PRM), are powerful tools for detailed quantification and have been used frequently in AD biomarker research^9,10^. However, these approaches are hypothesis-driven and are therefore useful only for measuring preselected proteins, minimising the likelihood of discovering novel biomarkers^11^. In contrast, the unbiased proteomic approach allows for the discovery of innovative biomarkers and development of new biological hypotheses of disease. However, the possibility that changes in proteins present in low quantities will remain undetected, and that different molecules with similar mass spectrometry (MS) signatures may not be distinguishable, are significant obstacles to the application of this technique in biomarker research^9,11^. Sequential window acquisition of all theoretical fragment ion spectra (SWATH)-based MS is a powerful and advanced proteomic technology that permits more precise identification of disease-specific changes in large protein pools. This tool can qualify and quantify proteins reliably and reproducibly at a large scale with deep proteome coverage^9,12,13^. In this study, we conducted a SWATH-based proteomic analysis to explore novel CSF biomarkers related to AD, and to determine whether proteomic changes can provide new insight into AD pathophysiology. Furthermore, we aimed to build new biomarker panels that can supplement Aβ- or tau-centred conventional biomarkers. To decrease the possibility of pathologies other than AD and age-related effects affecting our results, we recruited patients with early onset AD and age-matched controls after confirming the clinical diagnosis using CSF AD biomarkers^14^. We also screened for genetic mutations in *AβPP*, *PSEN1*, and *PSEN2*, and explored the impact of genetic variants on CSF proteomic analyte expression.

## Results

### AD-dependent changes in the CSF quantitative proteomic profile

The AD (n = 42) and control (n = 39) groups had similar demographic characteristics but significantly different clinical features, based on the Mini-Mental State Examination (MMSE), Clinical Dementia Rating Scale (CDR), CDR sum-of-boxes (SOB) scores, number of *APOE ε*4 alleles, medial temporal atrophy grades, and CSF Aβ42, tTau, and pTau181 levels (Table 1).

**Table 1:** Baseline data for the subjects included in this study.

	Control (n = 39)	AD (n = 42)	*p*-value
Sex (M:F)	10: 29	14: 28	0.476
Age at sampling (yo)	58.9 ± 6.3	60.3 ± 5.7	0.309
Education (y)	10.2 ± 3.2	10.2 ± 4.0	0.962
Duration of illness (y)	—	2.0 ± 1.2	
MMSE	28.3 ± 1.6	18.9 ± 6.4	<0.001
CDR	0 ± 0	1.1 ± 0.8	<0.001
CDR-SOB	0 ± 0.1	5.5 ± 5.3	<0.001
*APOE* ε4 carriers (%)	12.8	45.2	0.001
MTA visual grade^61^	0.1 ± 0.4	2.2 ± 0.9	<0.001
CSF Aβ42 (pg/mL)	704.2 ± 141.4	348.4 ± 88.5	<0.001
CSF tTau (pg/mL)	207.7 ± 55.3	637.8 ± 301.8	<0.001
CSF pTau181 (pg/mL)	42.2 ± 12.6	78.3 ± 20.1	<0.001

Values are shown in mean ± standard deviation.

*p-*values are determined through either independent t-test or *Chi*-squared tests depending on the character of the variables.

Abbreviations: AD, Alzheimer’s disease; *APOE*, apolipoprotein E; CDR-SOB, clinical dementia rating scale sum-of-boxes; CSF, cerebrospinal fluid; F, female; M, male; MTA, medial temporal atrophy determined by Schelten’s criteria in severe side^61^; MMSE, mini-mental state examination; y, years; yo, year-old.

**Figure 1:**
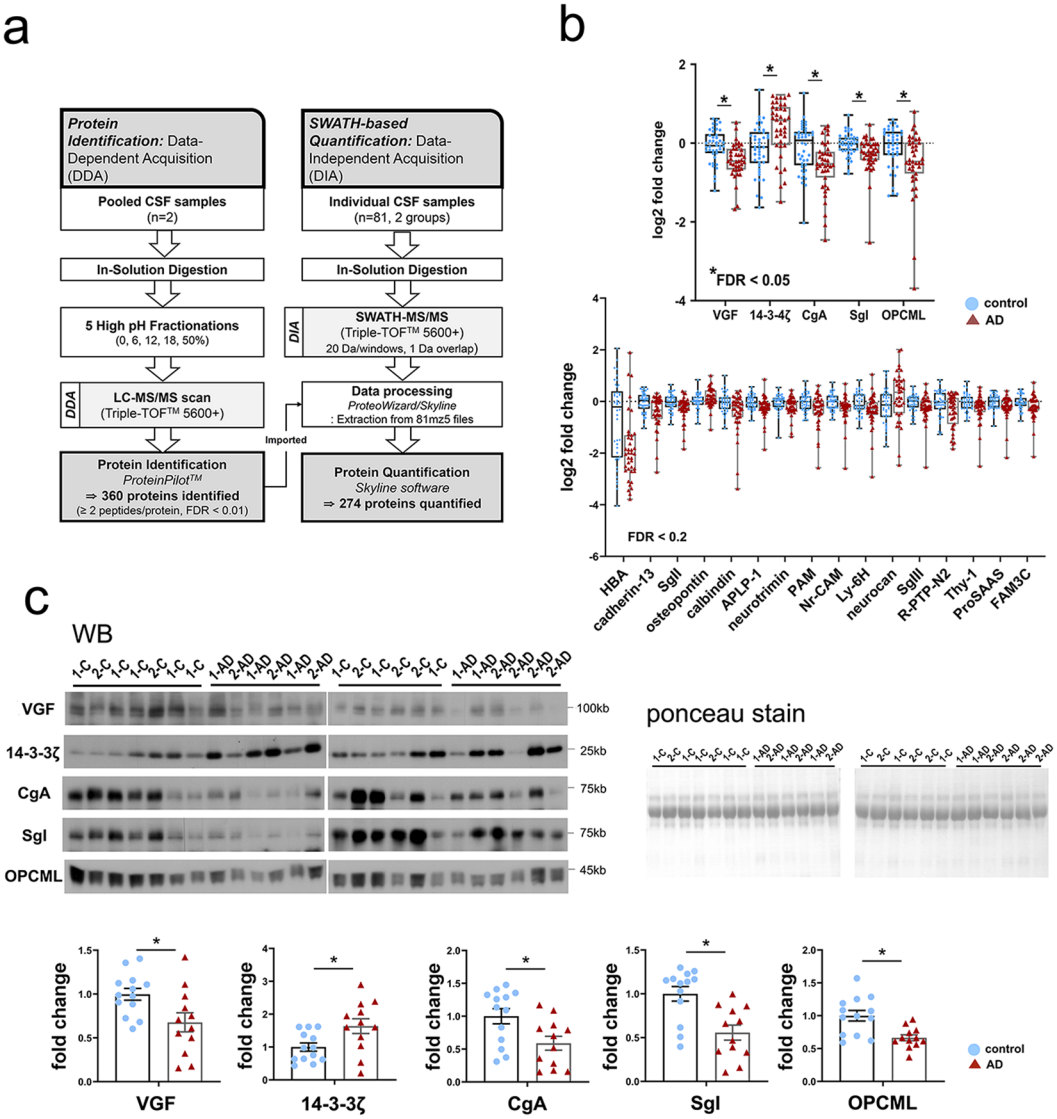
The results of SWATH–MS proteomic analysis and western blot validation. **(a)** Data-dependent acquisition (DDA) used pooled cerebrospinal fluid (CSF) samples to generate a CSF proteome library (left). Data-independent acquisition (DIA) was used to import DDA results to obtain the SWATH spectrum and quantify CSF proteins (right). **(b)** Box plots with scatter plots of log2 fold-change levels of CSF analytes revealing significant changes between AD and controls at *FDR < 0.05 (upper) and FDR < 0.2 (lower) on a *t* test with FDR correction. Box plots show the median and interquartile range; whiskers represent maximum and minimum values. (**c**) Western blot (WB) images and densitometric analyses show significant changes in indicated proteins in AD versus controls. The protein density in each lane was measured and normalized to total protein levels measured using Ponceau S staining of the corresponding polyvinylidene difluoride (PVDF) membrane. *p < 0.05 based on *t* test. Abbreviations: AD, Alzheimer’s disease; APLP-1, amyloid-like protein 1; C, control; CgA, chromogranin-A; SgI, secretogranin-1; FAM3C, protein FAM3C; FDR, false discovery rate; HBA, hemoglobin subunit alpha; Ly-6H, lymphocyte antigen 6 H; neurocan, neurocan core protein; Nr-CAM, neuronal cell adhesion molecule; OPCML, opioid-binding protein/cell adhesion molecule; PAM, peptidyl-glycine alpha-amidating monooxygenase; R-PTP-N2, receptor-type tyrosine-protein phosphatase N2; SgII, secretogranin-2; SgIII, secretogranin-3; Thy-1, Thy-1 membrane glycoprotein; WB, western blot; 14-3-3ζ, 14-3-3 protein zeta/delta; 1-, sample from the first cohort; 2-, sample from the second cohort.

Spectral libraries for the reference map were established through fractional analysis of two pooled CSF samples using liquid chromatography–tandem MS (LC–MS/MS)-based DDA. A total of 360 CSF proteins were identified by two or more peptides per protein at an accuracy of FDR < 0.01. On the entire sample (n = 81), we then performed high-resolution proteomic analysis using SWATH-based MS to identify all fragmented compounds in a systematic and unbiased manner, via DIA (Fig. 1a). A total of 274 proteins were quantified through SWATH-MS proteomics across all samples (see Supplementary Table S1). Statistical analyses revealed significant differences between the AD and control groups in the expression of five proteins with FDR < 0.05 and in the expression of another 16 proteins with FDR < 0.2 after we corrected for multiple comparisons using the FDR method^15,16^ (Fig. 1b; see Supplementary Table S2).

### Validation using western blot

We validated the AD-related changes in the top five candidate biomarkers using western blots of 25 CSF samples. Of these, five AD and eight control samples were from the cohort on which SWATH analysis was performed (the first cohort), whereas seven AD and five control CSF samples were from a different cohort (the second cohort). Consistent with the results of SWATH-MS, the protein expression of neurosecretory protein VGF, chromogranin-A (CgA), secretogranin-1 (SgI), and opioid-binding protein/cell adhesion (OPCML) decreased in AD samples, whereas that of 14-3-3 protein zeta/delta (14-3-3ζ) increased (Fig. 1c).

### Exploration of genetic impact on protein expression levels

Next, we explored whether rare genetic variants contributed to differential expression among the 21 analytes differentially expressed in patients with AD (FDR < 0.2). The frequency of variants with minor allele frequency (MAF) < 0.01 was determined via whole-exome sequencing, performed on all AD subjects (see Supplementary Fig. S1). Only rs377747918 in the *NCAN* gene had much higher frequency (7.1%; 3 of 42 patients) in AD subjects than in the general population (<1%). However, there was no difference in the fold-change levels of neurocan core protein measured through rs377747918 (1.0 ± 0.6 vs. 1.4 ± 0.9, *p* = 0.479). Therefore, the genetic variant did not appear to affect CSF protein expression levels in our subjects.

### Protein co-expression network and gene set enrichment analysis

**Figure 2:**
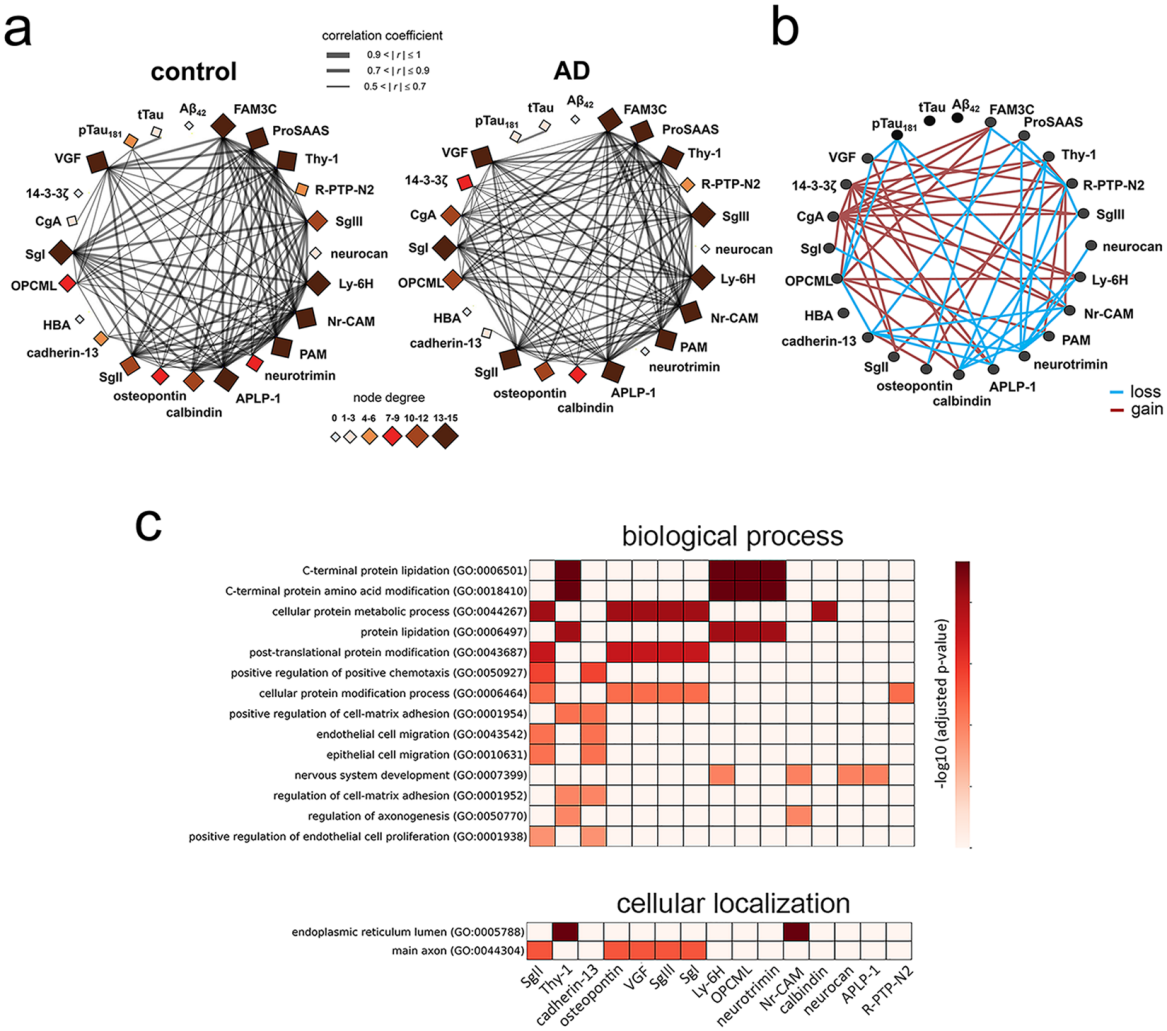
Co-expression network and gene-based enrichment analysis of CSF analytes. **(a)** Correlation maps demonstrate the interrelationships between fold-change levels of 21 SWATH–mass spectrometry (MS) proteins and CSF levels of Aβ42, tTau, and pTau181 (node), as determined by Pearson’s correlation followed by the Bonferroni correction to correct for multiple comparisons. Edge thickness is proportional to the strength of the correlation. **(b)** Gains (red) and losses (blue) of protein-to-protein interrelationships in the AD group compared to the control group are revealed by comparison of the left and right network maps in (**a**). **(c)** Gene-based enrichment analysis results for the top 21 CSF analytes; Gene Ontology (GO) biological processes (upper) and GO-cellular components (lower) are presented in order of adjusted significance, which we determined by genetic association using the EnrichR tool (http://amp.pharm.mssm.edu/Enrichr). Abbreviations: AD, Alzheimer’s disease; APLP-1, amyloid-like protein 1; CgA, chromogranin-A; SgI, secretogranin-1; FAM3C, protein FAM3C; HBA, hemoglobin subunit alpha; Ly-6H, lymphocyte antigen 6 H; neurocan, neurocan core protein; Nr-CAM, neuronal cell adhesion molecule; OPCML, opioid-binding protein/cell adhesion molecule; PAM, peptidyl-glycine alpha-amidating monooxygenase; R-PTP-N2, receptor-type tyrosine-protein phosphatase N2; SgII, secretogranin-2; SgIII, secretogranin-3; Thy-1, Thy-1 membrane glycoprotein; 14-3-3ζ, 14-3-3 protein zeta/delta.

To obtain system-level insight into the altered CSF proteomic profile in AD patients, co-expression analysis was performed on the 21 top proteins with FDR < 0.2 and on AD diagnostic biomarkers, including CSF Aβ42, tTau, and pTau181 proteins (see Fig. 2a and Supplementary Table S3). Several proteins were strongly co-expressed; more than half of these had ≥ 12 close interconnections (edges on the network) in the AD group. The network changed distinctly with AD diagnosis (Fig. 2b). The largest changes were seen in the 14-3-3ζ node (gain of nine edges with AD diagnosis), CgA (gain of eleven edges with AD), and neurotrimin (loss of eight edges with AD). None of the examined proteins exhibited a significant correlation with CSF Aβ42 or tTau levels, but the following proteins had co-expression relationships with pTau181 in the control group: VGF, OPCML, APLP-1, and Nr-CAM. These findings suggest that AD-associated changes in the CSF proteome among our subjects were independent of Aβ pathology, but linked to tau pathology.

The results of Gene Ontology (GO) enrichment analysis using the gene sets of the top 21 proteins demonstrated that post-translational modifications, including in C-terminal amino acids, lipidation, and cellular protein metabolic processes, were related to CSF proteomic changes (Fig. 2c). To a lesser extent, chemotaxis, cell migration, cell-matrix adhesion, and axonogenesis were enriched biological processes. The main functional locations of these proteins were the lumen of the endoplasmic reticulum and the main axon.

### Clinical utility of CSF analytes

**Figure 3:**
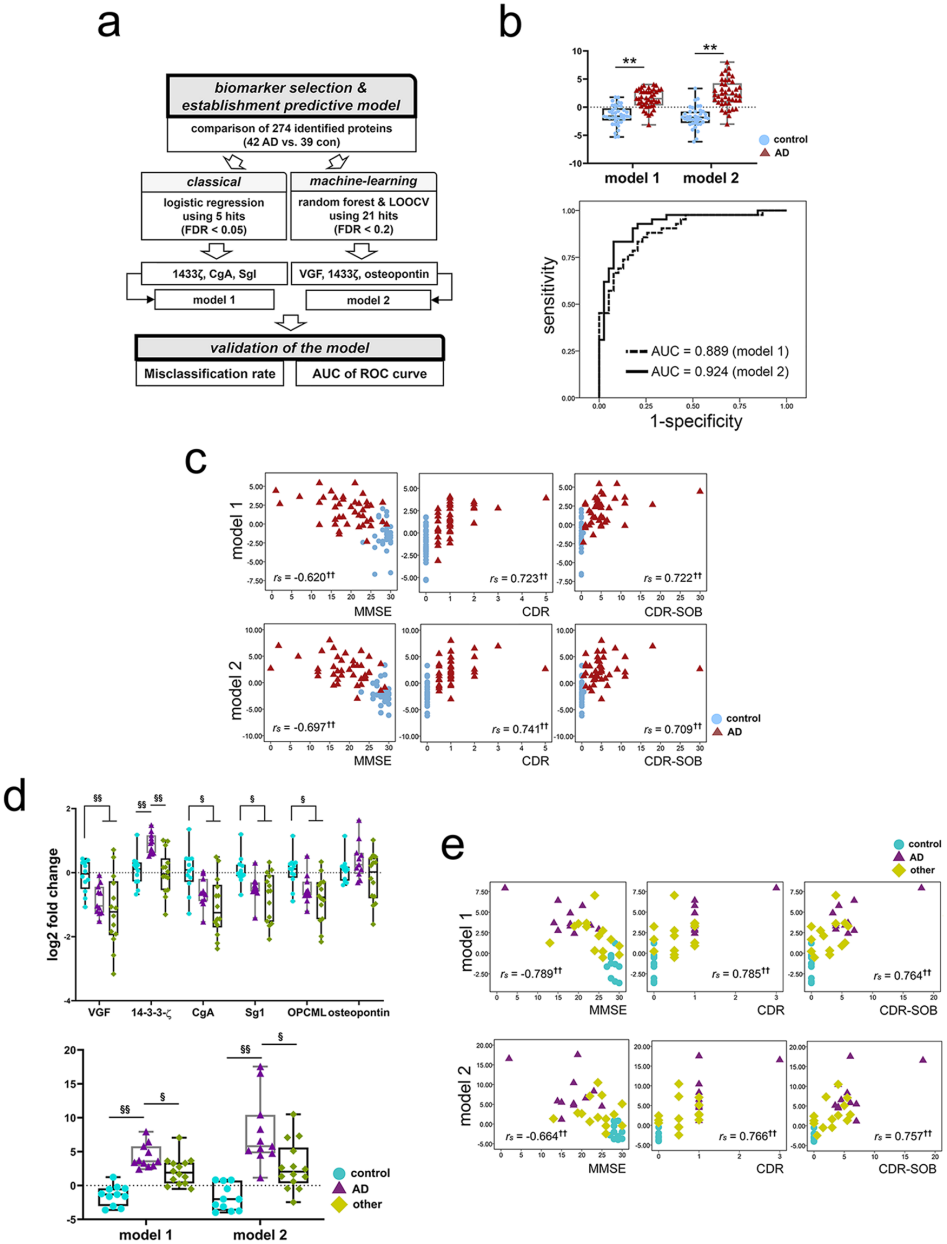
Analysis of the clinical utility of SWATH analytes. **(a)** The process of establishing CSF biomarker panels for AD through two different approaches: logistic regression (model 1) and random forest with leave-one-out cross-validation (LOOCV) (model 2). **(b)** A comparison of the values of model 1 and model 2 for the AD group vs. the control group (upper). The area under the curve in receiver operating characteristic (ROC) analyses of model 1 and model 2 for the diagnosis of Alzheimer’s disease. **p < 0.01 based on *t* test. **(c)** Correlations between CSF biomarker panel values and cognitive function (Mini-Mental State Examination, MMSE) and dementia severity profiles (Clinical Dementia Rating Scale [CDR], CDR sum-of-boxes [SOB] scores). *r*_*s*_: Spearman’s rank correlation coefficient, ^†^*p* < 0.05 and ^††^*p* < 0.01 based on Spearman’s rank correlation analysis. **(d)** A comparison of the values of six quantified proteins (log2 fold-change levels of each protein, upper) and CSF panels (equation values, lower) in three diagnostic groups from the second cohort. ^§^*p* < 0.05 and ^§§^*p* < 0.01 based on the Kruskal–Wallis test followed by pairwise post-hoc analysis with significance levels adjusted for the number of comparisons. **(e)** Correlations between CSF biomarker panel values and clinical profiles (MMSE, CDR, CDR-SOB). *r*_*s*_: Spearman’s rank correlation coefficient, ^†^*p* < 0.05 and ^††^*p* < 0.01 based on Spearman’s rank correlation analysis. Abbreviations: AD, Alzheimer’s disease; AUC, area under the curve; CDR-SOB, clinical dementia rating scale sum-of-boxes; CgA, chromogranin-A; FDR, false discovery rate; SgI, secretogranin-1; CON, control; MMSE, mini-mental state examination; OPCML, opioid-binding protein/cell adhesion molecule; 14-3-3ζ, 14-3-3 protein zeta/delta.

CSF expression of the top five analytes did not differ according to the presence of the *APOE *ε4 allele (*p* ≥ 0.05 overall). The fold-change in the top five CSF proteins according to AD diagnosis remained significant after we adjusted for age, education, sex, and the *APOE ε4* allele covariates in multivariate analyses (B = −4.489, *p* = 0.001 for VGF; B = 1.339, *p* = 0.021 for 14-3-3ζ; B = −2.326, *p* = 0.015 for CgA; B = −3.981, *p* = 0.015 for SgI; B = −1.818, *p* = 0.046 for OPCML). The ability of these proteins to distinguish between AD and controls was fair, with 0.678–0.752 area under the curve (AUC) in receiver operating characteristic (ROC) analysis (see Supplementary Fig. S2). To construct a more robust model, we performed a backward stepwise logistic regression analysis. We determined that 14-3-3ζ, CgA, and SgI were significant variables and that a regression equation for their combined value (3.639 + 3.530 × 14-3-3ζ − 3.384 × CgA − 5.222 × SgI: model 1) enhanced the diagnostic accuracy of the biomarkers (AUC = 0.889). Next, because the data mining approach had been better than logistic regression for establishing a diagnostic algorithm, we conducted a random forest analysis with cross-validation using leave-one-out cross-validation (LOOCV) with more analytes (21 proteins with FDR < 0.2; Fig. 3a)^17,18^. Each protein was ranked according to importance score and further validated through LOOCV (see Supplementary Fig. S2). The panel of 14-3-3ζ, osteopontin, and VGF was most effective at distinguishing AD patients from controls. Their combined value through the regression equation (− 1.49 + 2.70 × 14-3-3ζ + 7.02 × osteopontin − 10.01 × VGF: model 2) contributed greatly to an AD diagnosis (AUC = 0.924; Fig. 3b), with a lower misclassification rate than logistic regression (see Supplementary Fig. S3). Because osteopontin was newly recruited into the biomarker algorithm using a data-mining approach, its AD-related change in CSF samples from the first and second cohorts was validated with western blot (n = 13). The results differed significantly between AD and control samples (*p* < 0.05, Supplementary Fig. S4).

Spearman’s rank correlation analysis showed that both models correlated strongly with cognitive performance (*r*_*s*_ = − 0.620, *p* < 0.001, for model 1 and *r*_*s*_ = − 0.697, *p* < 0.001, for model 2 with MMSE) and dementia severity (*r*_*s*_ = 0.723, *p* < 0.001, for model 1 and *r*_*s*_ = 0.741, *p* < 0.001, for model 2 with CDR; *r*_*s*_ = 0.722, *p* < 0.001, for model 1 and *r*_*s*_ = 0.709, *p* < 0.001, for model 2 with CDR-SOB; Fig. 3c). The models performed better than any individual biomarker (see Supplementary Fig. S5).

### Validation using targeted PRM-MS in a different cohort including subjects with other brain disorders

We further validated candidate CSF biomarkers using high-resolution targeted PRM-MS in a different cohort (the second cohort) composed of subjects with other brain disorders (n = 14) in addition to AD (n = 11) and age-matched controls (n = 11; see Supplementary Table S4). The top five SWATH-MS analytes and osteopontin levels were quantified (see Supplementary Table S5), and their utility in combined algorithms was tested. Both CSF panels efficiently differentiated AD from other diseases and from controls (all *p* < 0.05; Fig. 3d). In addition, the panels had a strong persistent correlation with clinical profiles (Fig. 3e).

## Discussion

In the current study, we adopted strict diagnostic criteria to minimise age- and mutation-related confounding effects, and used unbiased high-resolution proteomic analysis to derive a new AD-related CSF protein profile and co-expression network. We also proposed a list of candidate AD biomarkers, demonstrating their utility in terms of AD diagnosis and their ability to reflect clinical severity.

Among the top five proteomic hits, VGF, CgA, and SgI are neurosecretory granin proteins. VGF is involved in the regulation of neurite growth, neurogenesis, and synaptic plasticity in the brain^19^; its synthesis is highly regulated by neurotrophic factors^20,21^. CgA and SgI are critical in the formation of secretory vesicles, and are involved in vasodilation, anti-apoptosis, microglial activation, neurotransmitter release, and synaptic function^22^. Altered CSF levels in VGF, CgA, and SgI have been suggested to represent synaptic loss and neuronal degeneration in AD patients^23–25^. Similarly, we identified significant correlations between the expression levels of these proteins and metrics of cognitive deterioration and dementia severity. However, the direction of change in the markers in our AD patients was contradictory to those reported in some previous studies^25^. This discrepancy might be due to dynamic changes in neurosecretory granin proteins at various stages of AD. A transient rise in the CSF levels of these synaptic proteins may occur as an early event in AD, followed by continual decreases along with disease progression^26^. This finding is supported by previous biological studies showing that a pathway compensating for synaptic damage increased the sizes of vesicles and synapses during the early stage of AD^27^.

Changes in the levels of these synapse-related proteins are not unique to AD; they have also been detected in other neurological disorders, such as multiple sclerosis^28^, schizophrenia^29^, Parkinson’s disease^30^, and amyotrophic lateral sclerosis (ALS)^31,32^. Consistent with these findings, we also found diminished CSF levels of VGF, CgA, and Sg1 in subjects with other brain disorders (e.g., frontotemporal dementia, Parkinson’s disease, and cerebrovascular disease). However, an optimal combination of biomarkers identified through multivariate analysis improved the accuracy of classification of AD versus other brain disorders.

Co-expression network analysis showed that the 14-3-3ζ protein had many co-expression relationships with other significant proteins in AD patients, the biological roles of which are known to be mainly related to synapse and neurite outgrowth^33–39^. The 14-3-3ζ protein had a unique power to discriminate between AD and other brain disorders (Fig. 3d). This common biomarker was incorporated into two biomarker panels that were obtained through different statistical approaches. The 14-3-3 family of proteins includes ubiquitous scaffolding proteins in the brain that regulate various signalling pathways by recognising phosphoserine or phosphothreonine^40^. In particular, the 14-3-3ζ protein plays a role as an effector during tau protein phosphorylation^41^ and regulates tau aggregation^42^. Enriched biological processes linked to AD-related proteomic changes in this study included post-translational protein modification, cell migration, and axonogenesis. Together, these results suggest that increased 14-3-3ζ protein levels in the brain^43^ enhance synaptic degeneration in AD patients via effects on tau pathology, which may be reflected in the expression changes observed in CSF synaptic proteins. However, the direct relationship between significant CSF protein changes and AD pathophysiology needs additional biological study, as we only evaluated proteins released into the CSF from brain tissue. Osteopontin was unique in that it was part of a diagnostic panel generated by the random forest method despite the fact that its fold-change in AD versus controls was moderate (FDR < 0.2). Osteopontin, a secreted glyco-phosphoprotein with a role in cell-matrix interactions and innate immunity, increases in AD patients during the acute phase of disease progression^44^. However, specificity to AD is unlikely given the results of our validation process and a previous report describing an increase in CSF osteopontin levels in LBD and PD in addition to AD^45^.

The new biomarker panels are thought to be better than the established CSF biomarkers with respect to clinical correlations of AD, but not in discriminating AD from control samples based on previous findings from our^14^ and other^46–50^ research groups. The new biomarkers demonstrated abundant interconnections and some connections with CSF pTau181 levels, which contrast to no co-expression with the Aβ42 protein. This result demonstrates the potential value of our candidate biomarkers as indicators of Aβ-independent pathology. The recent failure of Aβ-targeting therapy (despite evidence of effects on an Aβ-related biomarker)^51,52^ means that there is a need for additional biomarkers. Ideally, new biomarkers of Aβ- or tau-independent brain pathology should correlate with clinical symptoms^53^. In this context, the proteins (and combinations thereof) identified through SWATH–MS analysis in this study could be useful biomarkers for AD. The biological pathways related to post-translational protein modification, cell migration and axonogenesis are thought to synergistically enhance the protein–protein interactions involved in synaptic degeneration and brain dysfunction. The characteristic CSF protein profile described herein provides new targets for future biological research aimed at developing diagnostic tools for management of AD.

## Methods

### Participants

This study followed local clinical research regulations under the approval of the ethical review board of Soonchunhyang University Bucheon Hospital (SCHBC_IRB_2012-124) and Ajou University Medical Center (AJIRB-BMR-SMP-18-545). The study adhered to the principles of the Declaration of Helsinki. All participants and their legal guardians (in cases of dementia) gave written informed consent. Four university hospitals in the capital area of South Korea participated in this project and prospectively collected samples from January 2014 to January 2017 that were used for this study. The clinical diagnosis of AD was based on the National Institute on Aging and Alzheimer’s Association diagnostic guidelines^2^, incorporating information from a comprehensive neuropsychological battery^54^, laboratory and neurological examinations, and neuroimaging, which were performed within 1 month before collecting the CSF. Adherence to the CSF criteria for AD was considered to include AD subjects who met the diagnostic cut-offs of our laboratory^14^. When pathogenic AD mutations of *AβPP*, *PSEN1*, and *PSEN2* were identified, the subjects were excluded from the study. The age-matched control subjects had no history of a neurological disorder or systemic disorder that could potentially affect cognitive function, showed normal cognition on a neuropsychological test, and exhibited no abnormality on brain computed tomography or MRI. Moreover, CSF levels of Aβ42, tTau, and pTau181 in the normal range were mandatory in the control group. As blood contamination can affect CSF proteomic results, samples with >10/mm^3^ erythrocytes on a routine CSF analysis were excluded from the proteomic analysis in both groups. Clinical follow-up was performed at >6 months after the initial puncture to ensure the correct diagnosis. The second cohort, composed of subjects with various neurological disorders, was drawn from two university hospitals, and CSF samples were collected from January 2016 to June 2019. The basic requirements for participation were the same as those for the first cohort.

### CSF collection and preparation for analysis

CSF was sampled and stored according to the established protocol for a biomarker study^55^. CSF analyses were performed at a biomarker core laboratory. All CSF samples were thawed immediately prior to analysis. Aβ42, tTau, and pTau181 protein levels were measured using the INNOTEST enzyme-linked immunosorbent assay kit (Fuijrebio Diagnostics, Ghent, Belgium).

### SWATH-based MS

A DDA process with two pooled CSF samples from four individuals was executed to identify all fragmented compounds in a systematic and unbiased manner, and a CSF proteome library for SWATH–MS-based proteomic analysis was generated. Next we digested and prepared the CSF protein samples (n = 81) according to a previously described method for protein quantification by SWATH–MS^12,56^. The size of this study was comparable to a previous report on CSF^57^. A Triple-TOF 5600+ mass spectrometer (AB Sciex, Concord, ON, Canada) was used for all experiments. All spectra generated from DDA were searched using the ProteinPilot searching algorithm (SCIEX, Framingham, MA, USA) against a Uniprot human protein sequences database (UP000005640_9606_cRAP.fasta: total 21,159 protein entries) with the following search parameters: fully tryptic digestion; <50 ppm precursor ion tolerance; <0.5 Da fragment ion mass tolerance; fixed modifications for cysteine (+57 Da: carbamidomethylation); and biological modifications/artefacts, such as methionine oxidation (+16 Da). To reduce the false identification rate of proteins, we used a cut-off of two or more peptides as a qualification criterion, which permitted a peptide confidence level of >0.99. A CSF proteome spectral library was constructed using the Skyline software and the identified peptides^58^. All raw SWATH–MS data (WIFF files) were converted to the mz5 format using the ProteoWizard software, Version 3.0.6965 (http://proteowizard.sourceforge.net/). DIA data were extracted using the Skyline software, and DDA results were imported into Skyline with a cut-off criterion of 0.95. After extraction using the Skyline software, 274 proteins (1,006 peptides) were quantified in the 81 CSF samples. Two or more peptides were identified in all individual proteins, and 199 proteins had more than two peptides per protein. The areas under the peptide peaks for individual proteins were summed and used in expression analysis. Details of the SWATH–MS analysis are provided in Supplementary Information.

### Western blot

Western blot analysis of CSF was performed with 12 AD and 13 control samples. As a loading control, the expression of the target proteins was normalized to total protein levels measured using Ponceau S staining. Further details are available in the Supplementary Information.

### Targeted proteomics using PRM-MS

The sample preparation process was essentially the same as for the SWATH-MS analysis. The specifics of the process are described in the Supplementary Information. We targeted the top five proteins (FDR < 0.05) and osteopontin (FDR < 0.2) for PRM validation (see Supplementary Table S6). The latter was selected because of its contribution to the random forest model.

### Statistical analysis

All statistical analyses were performed using R statistical computing software (version 3.6.1; R Foundation for Statistical Computing, Vienna, Austria). As the raw value of SWATH-MS analysis is spectral area, which relies on the number of peptides identified from a given protein, direct comparison between proteins is inadequate. Therefore, the protein level fold-changes were calculated relative to the mean of control group in all subjects within a protein to perform a statistical analysis^59^. The independent-samples *t*-test and the Mann–Whitney *U* test were used to analyse differences between the AD and controls, with the choice of test depending on whether data were normally distributed. The Kruskal–Wallis test was performed for three-group comparisons in the second cohort. The χ^2^ test was applied for categorical variables. The Benjamini–Hochberg FDR method was used to correct for multiple comparisons^15,16^. Binary logistic regression was used to conduct multivariate analysis with various covariates. Logistic regression analysis with backward stepwise selection or the random forest method with LOOCV was used to establish a diagnostic CSF algorithm. ROC curve analysis and LOOCV were performed to validate the diagnostic accuracy of the biomarkers. Correlation analyses were conducted using Spearman’s rank correlation and Pearson’s correlation tests to estimate the relationships between the CSF analytes and elements of the clinical profile and to build a co-expression network, respectively. The Bonferroni correction was used to adjust for errors in co-expression network analyses due to multiple comparisons.

### Exome sequencing and calling of rare variants in gene sets of the SWATH analytes

Genomic DNA were extracted using the QIAamp® DNA blood midi kit (Qiagen, Valencia, CA, USA). Each exome library was prepared according to Agilent library preparation protocols (Agilent Human All Exon V5 + UTR kit; Agilent Technologies, Palo, Alto, CA, USA) and final libraries were then sequenced on an Illumina HiSeq. 2000 instrument (mean coverage × 200.3, 100-bp) by Macrogen (Seoul, Korea). To screen for effects of rare germline variants in 21 corresponding genes of CSF proteins with FDR < 0.2 on the expression changes in AD, the variants with MAF < 0.01 from the 1KG Database (East Asian) were checked against the open database of the Genome Aggregation Consortium (GnomAD; East Asian; http://gnomad.broadinstitute.org) and the Korean Reference Genome Database (KRGDB) from 1,722 Koreans (http://coda.nih.go.kr/coda/KRGDB/index.jsp). See the Supplementary Information for details.

### Gene set enrichment analysis

The enrichment analysis was performed using the updated EnrichR tool (http://amp.pharm.mssm.edu/Enrichr) to identify overrepresented GO biological processes and cellular components of the gene sets from the CSF analytes^60^.

## Supplementary information

Supplementary methods, Supplementary Figures.

Supplementary Tables.

## Data Availability

The proteomic datasets generated during this study are included in this published article as supplementary information files. The availability of the genetic datasets that support the findings of this study are not publicly available. Data are however available from the authors upon reasonable request and permission of institutional review board.
