# Supplementary material for: SWATH-MS analysis of cerebrospinal fluid to generate a robust battery of biomarkers for Alzheimer’s disease
Source: Sci Rep. 2020 May 4;10:7423. doi: 10.1038/s41598-020-64461-y (PMC7198522; doi:10.1038/s41598-020-64461-y)
Supplement: Supplementary file 1 — Supplementary methods, Supplementary Figures. [file 41598_2020_64461_MOESM1_ESM.doc]

**SWATH-MS analysis of cerebrospinal fluid to generate a robust battery of biomarkers for Alzheimer’s disease**

Sun Ah Park^1,2^*, Jin Myung Jung^3^, Jun Sung Park^4^, Jeong Ho Lee^4,5^, Bumhee Park^6,7^, Hyung Jun Kim^8^, Jeong-Ho Park^8^, Won Seok Chae^9^, Jee Hyang Jeong^10^, Seong Hye Choi^11^, Je-Hyun Baek^12^

**SUPPLEMENT INFORMATION**

**Supplementary methods**

**Supplementary figures**

(1) Supplementary Fig. S1. Diagram of overall process for calling rare germline variants in gene-sets of CSF analyte

(2) Supplementary Fig. S2. Biomarker selection for diagnostic algorithm using random forest and leave-one-out cross-validation

(3) Supplementary Fig. S3. Validation of two biomarker panels, model 1 and model 2

(4) Supplementary Fig. S4. Image of the western blot of osteopontin (left) and its densitometric analysis (right).

(5) Supplementary Fig. S5. The correlation analyses of the fold-change of top 5 SWATH-MS analytes and osteopontin with cognitive function (MMSE) and dementia severity (CDR and CDR-SOB).

**SUPPLEMENTARY DATASET** in a separate excel file:

(1) Supplementary Table S1. The full dataset of SWATH proteomics

(2) Supplementary Table S2. Group comparison of SWATH proteomic data in all identified proteins, control vs. Alzheimer’s disease

(3) Supplementary Table S3. The Pearson’s correlations analysis data of all the pairs of expression levels of 21 SWATH-MS analytes (FDR < 0.2) and Alzheimer’s disease biomarkers (Aβ_42_, tTau and pTau_181_)

(4) Supplementary Table S4. The baseline data of the subjects in the second cohort

(5) Supplementary Table S5. The dataset of targeted proteomic using PRM-MS in the second cohort

(6) Supplementary Table S6. The quantified peptide sequences of the target proteins for the parallel reaction monitoring-based proteomics

**Supplementary methods**

**SWATH-based mass spectrometry.** A data-dependent acquisition (DDA) process with two pooled CSF samples from four individuals was executed first to generate the CSF proteome library for the SWATH-MS-based proteomic analysis. Fifty μl of CSF sample was mixed in denaturing buffer (50 mM Tris-Cl, 1% sodium deoxycholate, 5 mM TECP) and incubated for 30 min at 60℃. Reduced thiol was alkylated using 10 mM iodoacetamide, and the reaction was quenched by applying 10 mM DTT for 30 min at 37℃. Dissolved organic carbon (DOC) was precipitated with 5% formic acid, and the digested peptide fractions were recovered from the supernatant after centrifugation (12,000× *g* for 10 min at 4℃). High pH reverse-phase fractionation (five fractions) was further applied to the digested protein samples. The dried peptide samples were resuspended in 100 mM ammonium formate buffer (pH 10), loaded onto a SPE C18 cartridge with 50 mM ammonium formate buffer (pH 10), washed with 1 mL of 50 mM ammonium formate buffer (pH 10), and then serially eluted with 1 mL of 50 mM ammonium formate buffer in acetonitrile solvent (0, 6, 12, 18, and 50%). After removing the DOC, all peptide samples were desalted on a 96-well Waters HLB cartridge, dried completely, and then stored prior to the liquid chromatography-tandem mass spectroscopy (LC-MS/MS) analysis. The CSF protein samples (n = 81) used for protein quantification by SWATH-MS were also digested and prepared according to the method described above. The Triple-TOF^TM^ 5600+ mass spectrometer (AB Sciex, Concord, ONT, Canada) was applied for all experiments as described previously^56^. In brief, the Eksigent NanoLC-2D+ with NanoFlex cHiPLC system (0.075-mm × 15-cm column) was coupled to the instrument to identify and quantify the CSF proteins. Solvent A contained 0.1% formic acid/H_2_O (v/v) in water, and solvent B was composed of 0.1% formic acid (v/v)/100% acetonitrile. The peptide samples were separated on an analytical column with a linear gradient of 2% to 35% solvent B over 30 min at a flow rate of 400 nL/min. The Chip nanoLC column was regenerated by washing with 60% solvent B for 50 min, followed by re-equilibration in 2% solvent B for 10 min. For the DIA experiment, the mass spectrometer was used with a survey scan (TOF-MS) for 250 ms, and then automated MS/MS scans were performed for 150 ms including the top 20 ions. The selection of parent ions for MS/MS was based on the criteria of more than 135 counts of precursor intensity with a charge state greater than 1 and the dynamic exclusion option (exclusion time: 15 sec). Operation of the mass spectrometer for protein quantitation by SWATH-MS was described previously^12^. A Triple-TOF^TM^ 5600+ instrument was used in looped product ion mode with 20-Da/mass windows (each SWATH window had a 1-Da overlap) covering the mass range of 400 to 1,000 Da. A set of 30 overlapping windows was constructed. Experiment 1: MS1 scan; experiment 2: 400–420 Da; experiment 3: 419–440 Da … experiment 31: 979–1000 Da. The proper collision energy for a two-charged ion centered on the window with a spread of 15 eV was adopted to determine the collision energy for an individual window. A 100-ms accumulation time was devoted to each fragment ion scan included in the initial survey scans. A total duty cycle of 3.1 s in high-sensitivity resolution mode was used for the MS/MS scan.

**Database searches.** All spectra generated from DDA were searched using the ProteinPilot^TM^ searching algorithm (SCIEX, Framingham, MA, USA) of the Uniprot human protein sequences database (UP000005640_9606_cRAP.fasta: total 21,159 protein entries) with the following search parameters: fully tryptic digestion, < 50-ppm precursor ion tolerance, < 0.5-Da fragment ion mass tolerance, fixed modifications for cysteine (+57 Da: carbamidomethylation), and biological modifications/artifacts such as methionine oxidation (+16 Da). To diminish the false identification rate of proteins, we used a cutoff of two or more peptides as a qualification criterion, which permitted a peptide confidence level > 0.99. A total of 360 proteins demonstrating two or more peptides per protein were identified using a modified Uniprot protein sequence database with a FDR < 0.01. A CSF proteome spectral library was constructed using Skyline software and the identified peptides^58^, which included redundant peptides with different charge states, retention times, and post-translational modifications.

**SWATH-MS data analysis.** All raw SWATH MS data (WIFF files) were converted to mz5 format using ProteoWizard software, Version 3.0.6965 (Palo Alto, CA, USA). The DIA data were extracted using Skyline software, and the DDA results were imported into Skyline, with a cutoff criterion of 0.95. The transition settings were as follows: MS1 filtering was performed for three isotope peaks in centroid mode with a 30-ppm accuracy; MS/MS filtering was used for the three isotope peaks in the centroid mode with 50-ppm accuracy. The retention time window was considered within 10 min of the MS/MS identification period, and the extracted data were manually confirmed considering the retention time and rank order of peak intensities in both the library and SWATH spectra. After extraction using Skyline software, 274 proteins (1,006 peptides) were quantified in the 81 CSF samples. Two or more peptides were identified in all individual proteins, and 199 proteins had more than two peptides per protein. Normalization was performed only upon sample volume, which allows the best discrimination results among sample groups. Quantification error was confirmed below 11.2% in three replicates for 100 CSF proteins.

**Western blot analysis and total protein staining.** Twenty μg of CSF protein was loaded and electrophoresed on 8 - 12% SDS polyacrylamide gel and transferred onto polyvinylidene difluoride (PVDF) membranes. The membranes were blocked with 5% non-fat milk in TBST (150 mM, 10 mM Tris–HCl, and 0.1% Tween 20 at pH 7.4) for 1 hour and then incubated overnight with the primary antibodies of VGF (mouse monoclonal Ab, #sc-365397, Sigma-Aldrich), 14-3-3 beta/epsilon/zeta (pan 14-3-3 mouse monoclonal Ab, #MA1-34561, ThermoFisher Scientific), CgA (rabbit polyclonal Ab, #ab15160, Abcam), SgI (rabbit polyclonal Ab, ab12242, Abcam), and OPCML (goat polyclonal Ab, #AF2777, R&D Systems) in a blocking buffer (1:1,000) overnight. After washes with TBST, bound primary antibodies were detected with corresponding horseradish peroxidase (HRP)-conjugated secondary antibodies (1:10,000; Santa Cruz Biotechnology) for 1 hour. The blots were visualized using enhanced chemiluminescence substrate (Bio-Rad, Hercules, CA, USA). The respective protein band intensity was quantified by densitometric analysis using the NIH ImageJ ver. 2.3 (NIH). Total protein staining was performed on the PVDF membrane after western blot using ready-to-use Ponceau S stain solution (#K793, VWR life science) following the manufacturer’s instructions. The protein density in each lane of the stained membranes was measured using ImageJ ver2.3, and was used as the loading control.

**Parallel Reaction Monitoring (PRM)-based target proteomics.** A PRM-based MS was performed for the target protein quantification. Sample preparation process was same as SWATH-MS analysis. The 15 precursor’s m/z for 18 peptides from six proteins were selected for PRM-MS (4 m/z isolation width, 50msec, Resolution = 7500, fixed CE = 28). Thermo Q-Exactive HF-X mass spectrometer (Bremen, Germany) was applied for all PRM analysis. Thermo Scientific Dionex Ultimate^TM^ 3000 UPLC system (0.075-mm × 70-cm column) was coupled to the instrument in order to quantify the CSF proteins. Solvent A contained 0.1% formic acid/H2O (v/v) in water, and solvent B was composed of 0.1% formic acid (v/v)/100% acetonitrile. The peptide samples were loaded on trap column (0.050 mm × 2 cm column) for 8 min at a flow rate of 5 μL/min and then separated on an analytical column with a linear gradient of 5% to 10% solvent B over 1 min and 40% solvent B over 32 min at a flow rate of 350 nL/min. The column was regenerated by washing with 80% solvent B for 2 min, followed by re-equilibration in 5% solvent B for 17 min. The 15 inclusion lists for the PRM analysis are as following: 436.88, 454.27, 510.26, 541.28, 549.28, 595.33, 662.82, 673.82, 692.33, 706.33, 709.863, 729.37, 733.68, 778.37, 1098.48 m/z.

**PRM-MS data analysis.** The software (e.g. Skyline), retention time window, and normalization was used as the same to the data processing for SWATH-MS analysis. The transition settings were as follows: MS1 filtering was performed for three isotope peaks in Orbitrap mode with a 60,000 resolution accuracy; MS/MS filtering was used for the three product ion peaks in the Orbitrap mode with a 7,500 resolution accuracy. The retention time window was considered within 10 min of the MS/MS identification period, and the extracted data were confirmed considering the retention time and rank order of peak intensities in both the library and PRM spectra. After extraction using Skyline software, the six proteins (16 peptides) were relatively quantified among 36 CSF samples (control, AD, and other diseases).

**Exome sequencing and calling of rare variants in gene sets of the SWATH analytes.** Genomic DNA were extracted using the QIAamp® DNA blood midi kit (Qiagen, Valencia, CA, USA) following the manufacturer’s instructions. Each exome library was prepared according to Agilent library preparation protocols (Agilent Human All Exon V5+UTR kit; Agilent Technologies, Palo, Alto, CA, USA) and final libraries were then sequenced on an Illumina HiSeq2000 instrument (mean coverage × 200.3, 100-bp) by Macrogen (Seoul, Korea). Reads were mapped to GRCh38 as a reference genome using BWA 0.7.13. Picard Tools 2.1.0 was used to flag duplicate reads. We applied GATK to realign the indels, recalibrate the base quality score, and detect single nucleotide polymorphisms (SNPs) and indels using the Haplotype caller across all AD samples simultaneously according to GATK Best Practices 3.5 (https://software.broadinstitute.org/gatk/best-practices/). Finally, we annotated joint variant calling files with the Variant Effect Predictor from Ensemble (release 90). To screen for effects of rare germline variants in 21 corresponding genes of CSF proteins with FDR < 0.2 on the expression changes in AD, the variants with MAF < 0.01 from the 1KG Database (East Asian) were checked against the open database of the Genome Aggregation Consortium (GnomAD; East Asian; http://gnomad.broadinstitute.org) and the Korean Reference Genome Database (KRGDB) from 1,722 Koreans (http://coda.nih.go.kr/coda/KRGDB/index.jsp). In addition, the pathogenicity of rare variants was subsequently filtered when these satisfied any of following criteria: Condel (“Deleterious”), SIFT (“Deleterious”), PolyPhen (“Damaging”), LoFTool (< 0.001), MaxEntScan (△ < −0.15).

**Supplementary Fig. S1.** Diagram of overall process for calling rare germline variants in gene-sets of CSF analyte.


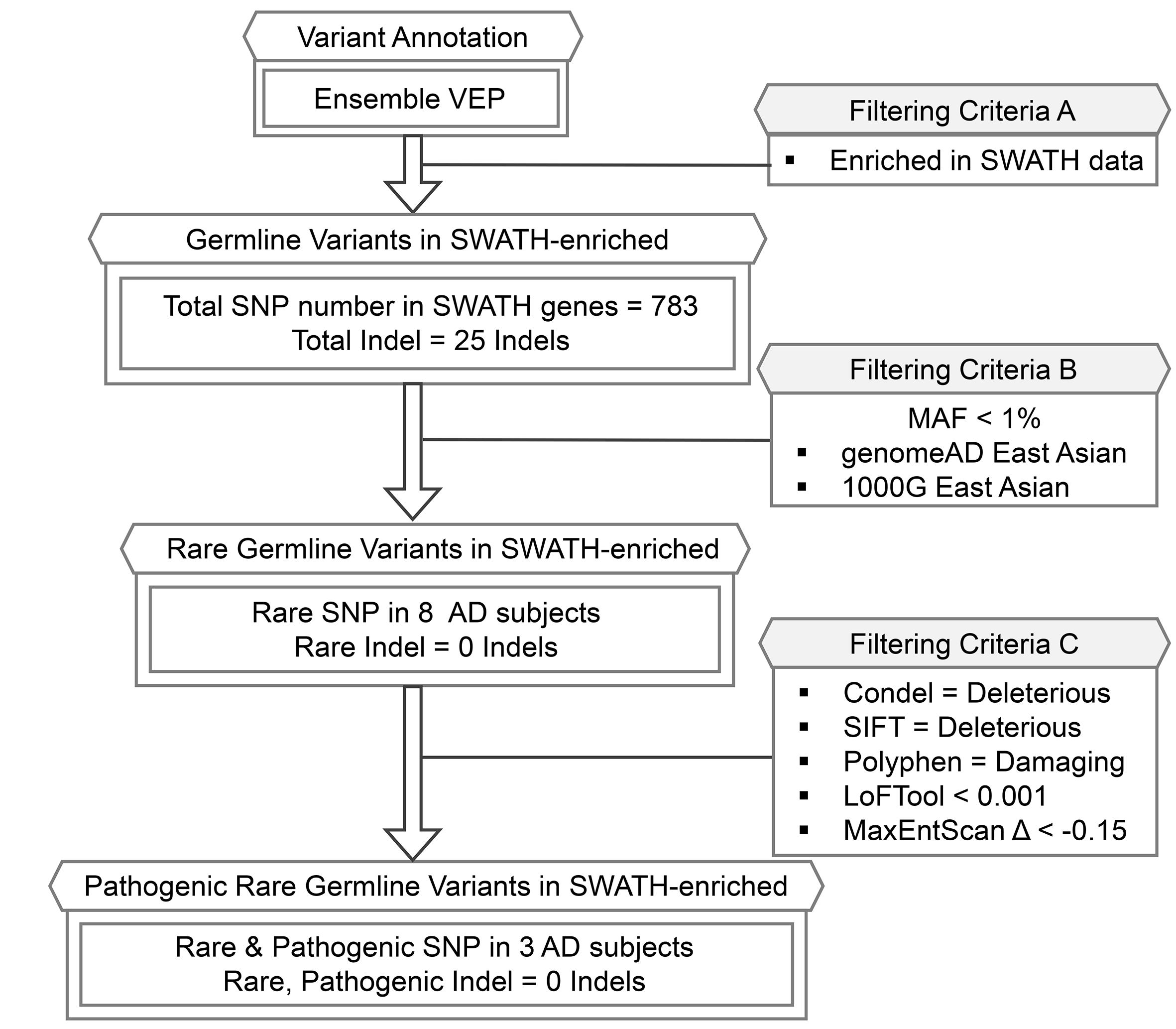


Abbreviations: Condel, Consensus deleteriousness score of non-synonymous single nucleotide variants; AD, Alzheimer’s disease; Indel, insertion or deletion of bases; MAF, minor allele frequency; Polyphen, polymorphism phenotyping; SIFT, sorting intolerant from tolerant; SNP, single nucleotide polymorphism; SWATH, sequential window acquisition of all theoretical fragment ion spectra; VEP, variant effect predictor

**Supplementary Fig. S2.** Biomarker selection for diagnostic algorithm using random forest and leave-one-out cross-validation (LOOCV). **(a)** Area under the curve (AUC) on receiver operating characteristic (ROC) analysis of individual top 21 SWATH-analytes. **(b)** Importance ranking of the individual biomarker by random forest. **(c)** Plot of accuracy for the model using LOOCV. The optimum (highest value on y-axis) is found on the 3-protein combination (corresponding value on x-axis).


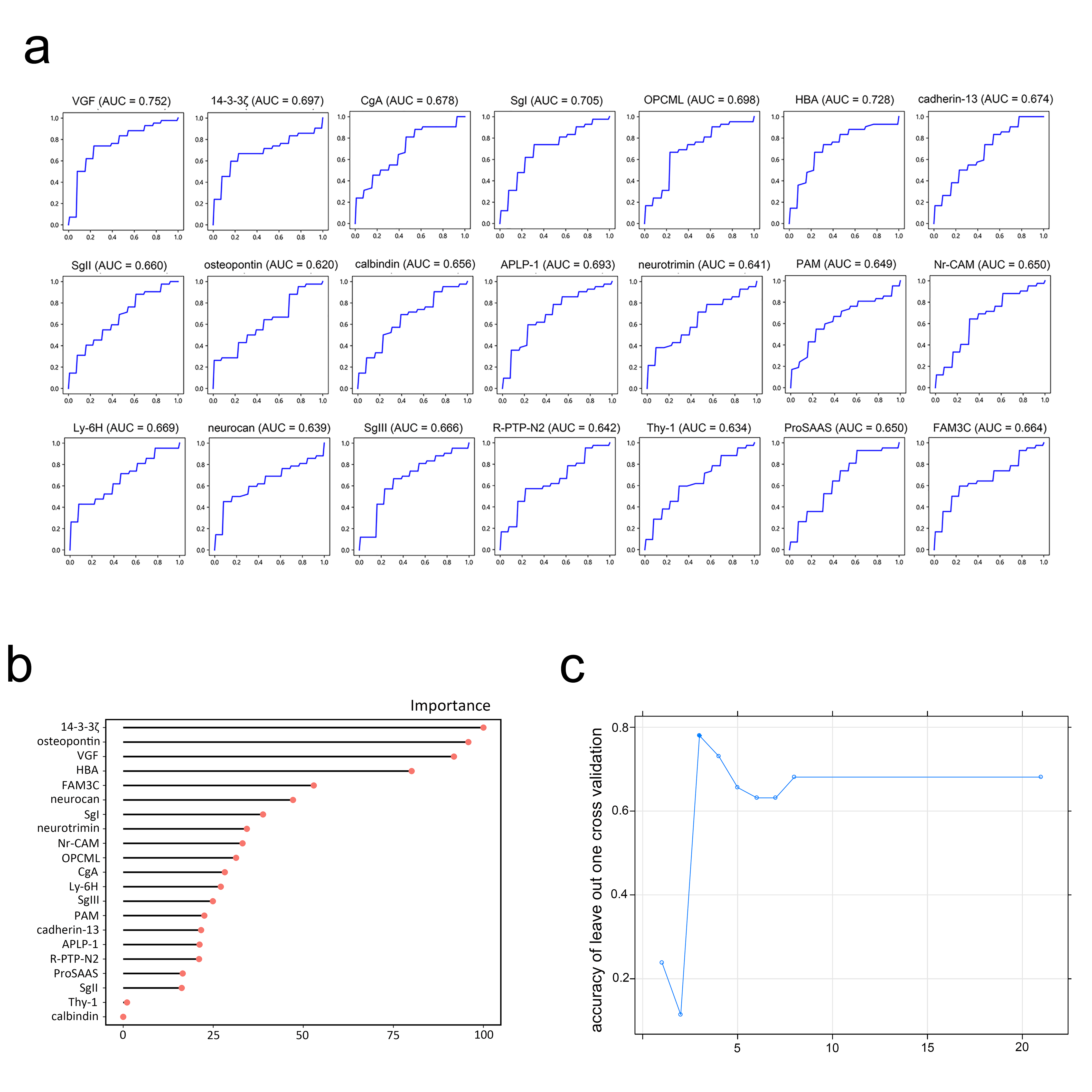


Abbreviations: APLP-1, amyloid-like protein 1; AUC, area under the curve; CgA, chromogranin-A; SgI, secretogranin-1; FAM3C, protein FAM3C; HBA, hemoglobin subunit alpha; Ly-6H, lymphocyte antigen 6H; neurocan, neurocan core protein; Nr-CAM, neuronal cell adhesion molecule; OPCML, opioid-binding protein/cell adhesion molecule; PAM, peptidyl-glycine alpha-amidating monooxygenase; R-PTP-N2, receptor-type tyrosine-protein phosphatase N2; SgII, secretogranin-2; SgIII, secretogranin-3; Thy-1, Thy-1 membrane glycoprotein; VGF, neurosecretory protein VGF; 14-3-3ζ, 14-3-3 protein zeta/delta

**Supplementary Fig. S3.** Validation of two biomarker panels, model 1 and model 2. **(a)** Receiver operating characteristic (ROC) curve analysis of two biomarker algorithms and their components. **(b)** Histogram demonstrating classification accuracy of two biomarker panels.


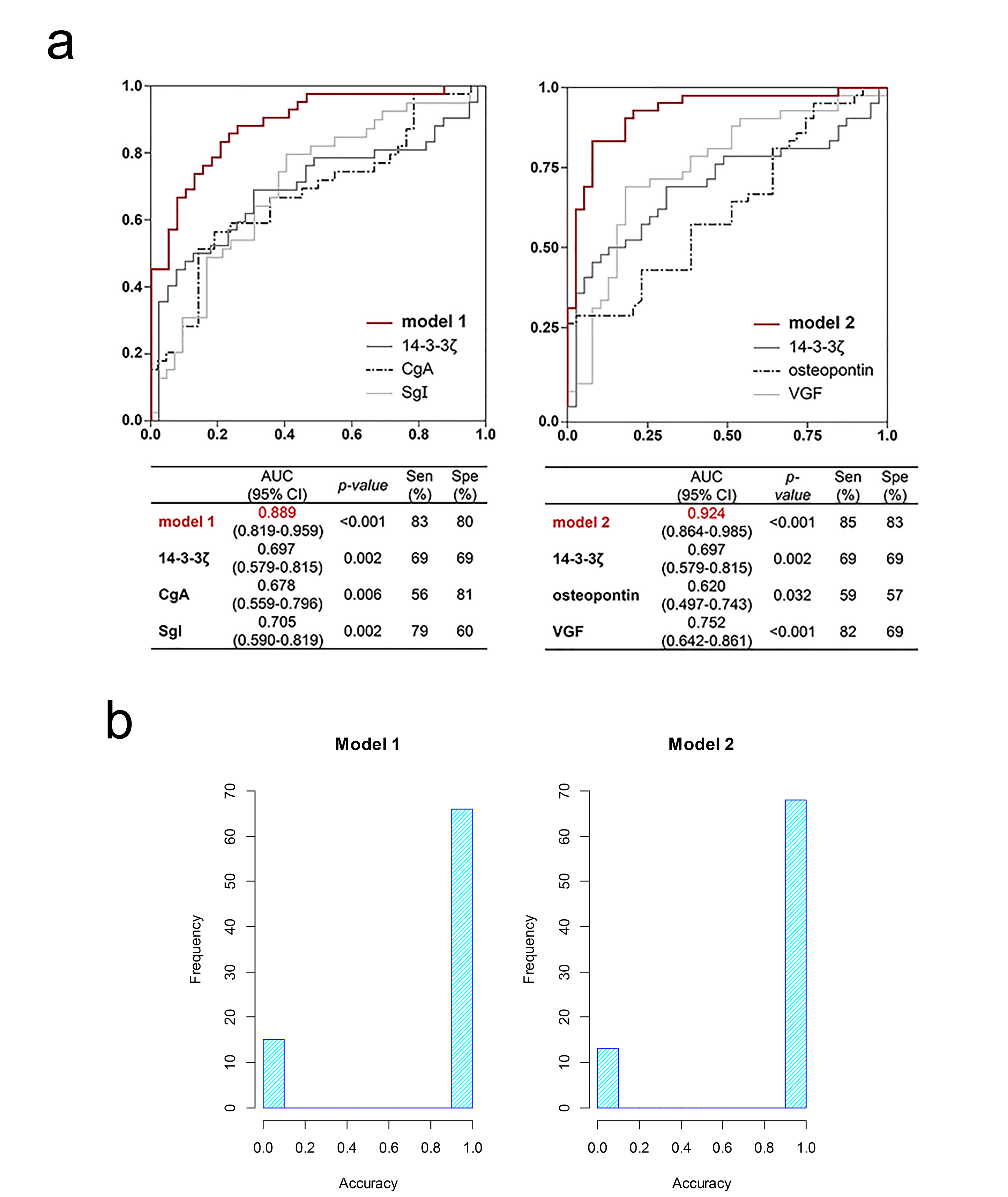


Abbreviations: AUC, area under the curve; CgA, chromogranin-A; Sg-I, secretogranin-1; Sen, sensitivity; Spe, specificity; VGF, Neurosecretory protein VGF; 14-3-3ζ, 14-3-3 protein zeta/delta

**Supplementary Fig. S4.** Image of the western blot of osteopontin (left) and its densitometric analysis (right). The protein band intensity was normalized to total protein expression based on Ponceau S staining. Subsequently, the fold-change in osteopontin expression in the AD group compared to the control group was calculated and statistically analysed.


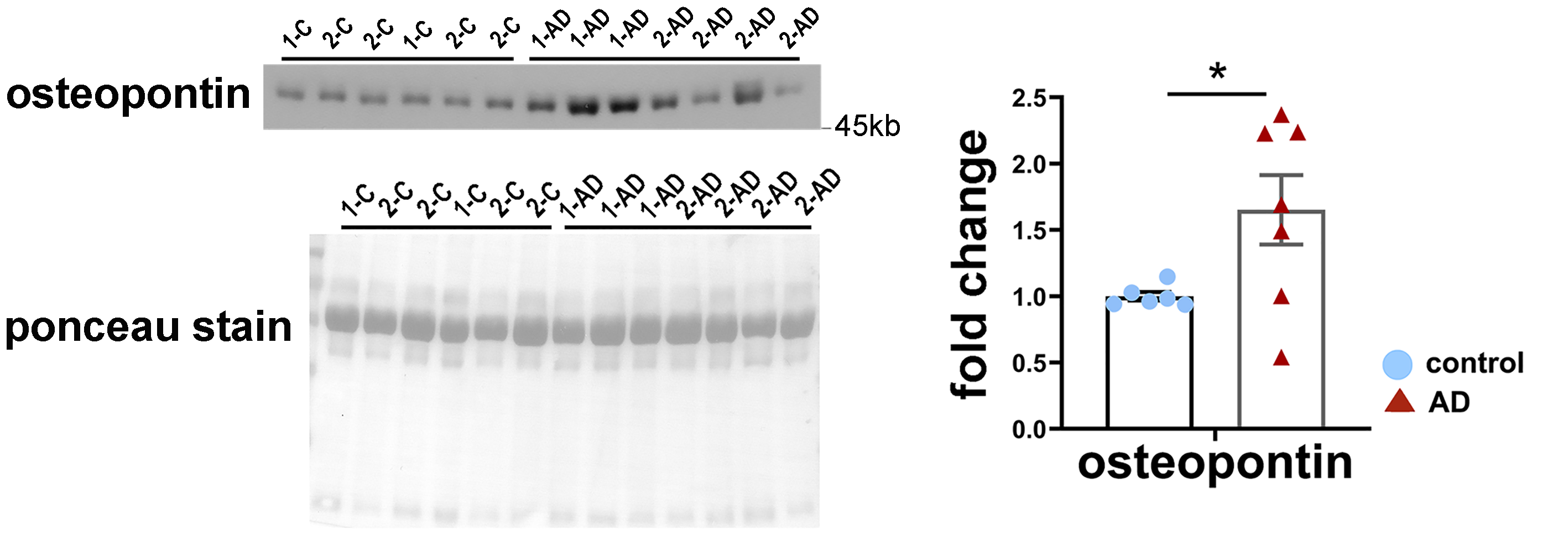


AD, Alzheimer’s disease; C, control; 1-, sample from cohort 1; 2-, sample from cohort 2

******p* < 0.05 based on the *t*-test, AD *vs.* control.

**Supplementary Fig. S5.** The correlation analyses of the fold-change of top 5 SWATH-MS analytes and osteopontin with cognitive function (MMSE) and dementia severity (CDR and CDR-SOB).


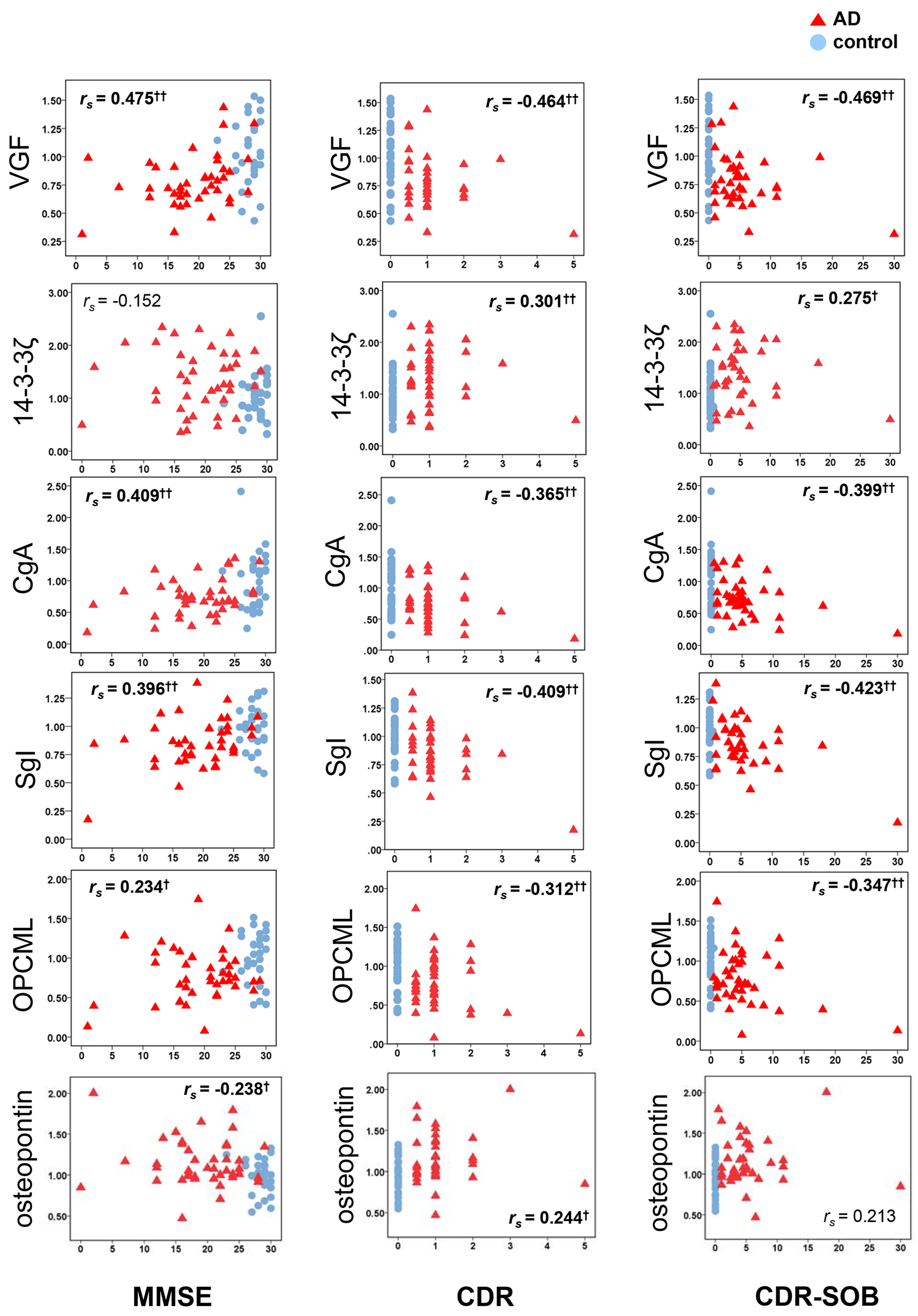


Abbreviations: AD, Alzheimer’s disease; CDR-SOB, clinical dementia rating scale sum of boxes; CgA, chromogranin-A; Sg-I, secretogranin-1; MMSE, mini-mental state examination; OPCML, opioid-binding protein/cell adhesion molecule; VGF, Neurosecretory protein VGF; 14-3-3ζ, 14-3-3 protein zeta/delta

*r_s_*: Spearman’s rank correlation coefficient

^†^*p* < 0.05 and ^††^*p* < 0.01 based on Spearman’s rank correlation analysis.
